# Beyond Sequencing: Integrating MLPA Reveals Hidden Structural *PKD2* Variants and Enhances Mutation Detection in a Highly Selected ADPKD Greek Cohort

**DOI:** 10.3390/medicina62091771

**Published:** 2026-09-15

**Authors:** Akrivi Papachristopoulou, Angeliki Margoni, Aimilios Christos Simoudis, Aristea Mylona, Olga-Irini Kroupi, Petros Nikolopoulos, Athanasios G. Papavassiliou, Demetrios V. Vlahakos, Christos Kroupis

**Affiliations:** 1 Department of Clinical Biochemistry, Attikon University General Hospital, Medical School, National and Kapodistrian University of Athens, 12462 Athens, Greece; 2 Department of Biological Chemistry, Medical School, National and Kapodistrian University of Athens, 11527 Athens, Greece; 3 Nephrology Unit, 2nd Department of Internal Medicine, Attikon University General Hospital, Medical School, National and Kapodistrian University of Athens, 12462 Athens, Greece

**Keywords:** late-onset ADPKD, genetic mutations, *PKD2* variants, phenotype-based diagnostic algorithm, MLPA

## Abstract

*Background and Objectives*: The genetic architecture of late-onset autosomal dominant polycystic kidney disease (ADPKD) remains incompletely defined. Pathogenic mutations in two genes that encode polycystin proteins, *PKD1* and *PKD2*, prevail among these patients. Detection of *PKD1* alterations by a sequencing method is challenging as this is a large gene with high GC content and multiple pseudogenes. Although next-generation sequencing (NGS) has become the cornerstone of molecular diagnosis, its limited availability in several settings and its limited sensitivity for structural variants may create a diagnostic blind spot, particularly for *PKD2*. Because *PKD2*-associated ADPKD follows a milder clinical course with delayed progression to end-stage renal disease (ESRD), its contribution to late-onset disease may be systematically underestimated. We hypothesized that a phenotype-driven strategy integrating copy number analysis would uncover clinically relevant *PKD2* variants overlooked by DNA sequencing methods. *Materials and Methods*: Six well-characterized consecutive ADPKD patients that entered KRT (kidney replacement therapy) above 70 years old underwent targeted *PKD2* analysis using bidirectional Sanger DNA sequencing of all coding exons and exon–intron boundaries, complemented by multiplex ligation-dependent probe amplification (MLPA) for copy number variant detection. *Results*: Pathogenic *PKD2* variants were identified in 33% of the patients in this limited cohort. These included the recurrent nonsense variant p.Arg872Ter and a previously undescribed multi-exonic deletion encompassing exons 1–9 that was detectable exclusively by MLPA. Additionally a novel variant of unknown significance (VUS) (p.Leu273Gln) was detected. The high diagnostic yield in this phenotypically highly enriched cohort highlights the value of targeted structural variant analysis. *Conclusions*: Our findings identify structural *PKD2* variation as an under-recognized cause of genetically unresolved late-onset ADPKD and demonstrate that phenotype-guided incorporation of copy number analysis can overcome limitations of NGS alone. A combined sequencing–MLPA approach may therefore provide a more complete and clinically informative molecular diagnosis, particularly in carefully selected patients with late-onset ADPKD.

## 1. Introduction

Autosomal dominant polycystic kidney disease (ADPKD), the most common monogenic cause of kidney failure worldwide, is characterized by substantial heterogeneity in disease penetrance, clinical expression, and long-term outcomes, reflecting its complex genetic architecture, which predominantly involves variants in *PKD1* (~80%) and *PKD2* genes [1,2,3,4,5,6]. The burden of ADPKD is unequivocal: more than 50% of affected individuals progress to end-stage renal disease (ESRD), accompanied by a 1.6- to 3.2-fold increase in mortality compared with the general population [4,7,8,9]. Failure to recognize disorders that mimic the phenotype of ADPKD but follow distinct inheritance patterns, pathogenic mechanisms, and clinical trajectories may result in misdiagnosis, inappropriate management, inadequate surveillance, and ultimately poorer clinical outcomes [10,11,12].

Persistent elevations in cyclic adenosine monophosphate (cAMP) and activation of protein kinase A (PKA)-dependent pathways promote epithelial proliferation and fluid secretion, while progressive cyst expansion induces chronic hypoxia, inflammation, oxidative stress, and fibrotic remodeling, collectively creating a microenvironment that facilitates cystogenesis and on rare occasions tumorigenesis [4,6,7,8,9,10,11,12]. These observations underscore the need for a multidimensional investigation of ADPKD pathobiology through the lens of molecular diagnostics in the era of precision medicine.

Despite significant advances in next-generation sequencing (NGS), molecular diagnosis remains challenging, particularly for *PKD1*, owing to its large genomic size, high GC content, and extensive homology with multiple pseudogenes. Furthermore, NGS-based approaches may inadequately detect structural variants, potentially contributing to underdiagnosis, particularly in older patient populations [1,2,3,4,5,6]. To address these limitations, we emphasize the integration of Sanger sequencing of coding regions and exon–intron boundaries with multiplex ligation-dependent probe amplification (MLPA) for comprehensive copy number variant detection.

Taken together, contemporary ADPKD assessment should integrate demographic and clinical variables (age, sex, recurrent or persistent hematuria) with functional and structural disease markers, including estimated glomerular filtration rate (eGFR), albumin-to-creatinine ratio (ACR), and height-adjusted total kidney volume (ht-TKV) measured by magnetic resonance imaging (MRI), interpreted through validated imaging classification systems [7,8,9] (https://www.mayo.edu/research/documents/classification-of-typical-adpkd-calculator/doc-20094754 (accessed on 30 June 2026)).

From a genetic perspective, variants in *PKD1* and *PKD2* account for approximately 90% of genetically resolved cases and define the principal disease subtypes. Pathogenic alterations in *PKD1 gene*, located on chromosome 16 and responsible for approximately 78–80% of cases, are generally associated with a more severe clinical phenotype through disruption of polycystin-1-mediated signaling pathways, including mTOR and MAPK cascades. In contrast, *PKD2* mutations (~12–15% of cases) impair polycystin-2-dependent calcium signaling and are typically associated with attenuated disease expression, slower cyst growth, and delayed progression to ESRD [1,2,3,4,5,6,7,13,14,15]. Although NGS-based panels increasingly incorporate additional cystic disease genes, e.g., Intraflagellar Transport 140 (*IFT140*), Dolichyl-Phosphate β-Glucosyltransferase (*ALG5*), Alpha-1,2-Mannosyltransferase (*ALG9*), DnaJ Heat Shock Protein Family (Hsp40) Member B11 (*DNAJB11*), Glucosidase II Alpha Subunit (*GANAB*), and NIMA-Related Kinase 8 (*NEK8*), their implementation remains variable across clinical settings, especially in countries with limited NGS resources, and surveillance paradigms continue to rely largely on conventional clinical parameters [9,10,11,12,13,14,15,16]. Genetic heterogeneity therefore remains a cornerstone of contemporary disease stratification.

Current KDIGO guidelines do not recommend routine genetic testing for the diagnosis of ADPKD in all patients. Rather, testing is suggested primarily in cases of very-early-onset disease, atypical or equivocal clinical presentations, or cystic kidney disease occurring in the absence of a positive family history, where a de novo pathogenic variant is suspected [16,17]. Nevertheless, delineation of the underlying genetic substrate remains fundamental for meaningful risk stratification, enabling classification into low-, intermediate-, and high-risk trajectories through consideration of both major disease-causing and modifier genes [5,17,18,19,20,21,22,23]. In this regard, the PROPKD score (https://adpkdsim.org/expert/prognostic-tools/propkd-score (accessed on 30 June 2026)) incorporates genetic information into prognostic modeling, assigning differential weight to *PKD2* variants, *PKD1* missense variants, and *PKD1* truncating mutations to estimate the age at which kidney replacement therapy, including transplantation, hemodialysis, or peritoneal dialysis, is likely to be required.

Within this framework, the present pilot study aimed to evaluate the clinical relevance of *PKD2* variant detection in a highly selected subset of patients with ADPKD characterized by late-onset ESRD and to support the implementation of a pragmatic and cost-effective diagnostic strategy integrating Sanger sequencing with MLPA to optimize molecular diagnosis and improve genetic characterization.

## 2. Materials and Methods

### 2.1. Study Design and Population

For this pilot study, samples were collected from 10 subjects as follows: six were consecutive ADPKD patients who either themselves entered kidney replacement therapy (KRT) after 70 years old or were worried about the prognosis and had a parent family member entering KRT after 70 years old, two were known *PKD1* mutation carriers (p.Phe1416LeuFsTer16 and p.Phe2043_Ala2045del) and two were healthy subjects free of renal cysts and CKD. The age selection criterion was applied after carefully reviewing literature regarding kidney survival in *PKD2* carriers (in the recent Lavu et al. study, median age for PKD2 males was 71.2 years [21]; a rounded simple age number was selected).

The study was initiated after approval from the “Attikon” General University Hospital Scientific and Bioethics committee (75/12-2-2020) and was conducted in compliance with the principles of the National and Kapodistrian University of Athens. In order to collect samples and medical data, patients’ signed informed consents were obtained.

### 2.2. DNA Isolation–DNA Sequencing

Peripheral blood samples were collected in EDTA tubes and genomic DNA extraction was performed using the Blirt ExtractMe DNA Blood Kit (Blirt, Danzig, Poland) according to the manufacturer’s protocol. DNA yield and purity were assessed fluorometrically using the Qubit dsDNA High Sensitivity Assay (Thermo Fisher Scientific, Waltham, MA USA), ensuring optimal concentration and minimal protein contamination (A260/280 ratio ~1.8). All 15 exons of the *PKD2* gene, including flanking intronic regions, were amplified by polymerase chain reaction (PCR) with BioMix Red polymerase (Bioline, UK). Primer sets were selected from Deltas (2001) except from those for exons 13 and 14 [24,25]. All PCRs were performed simultaneously by using the gradient feature of the Eppendorf 5331 Mastercycler, (Eppendorf, Hamburg, Germany) with annealing temperatures tailored to each primer pair to minimize nonspecific amplification (range 66–69 °C). Amplicon integrity and size specificity were verified by agarose gel electrophoresis (1.5–2%), followed by visualization under UV illumination after ethidium bromide staining.

PCR products were subsequently purified to remove primers, nucleotides, and enzymes using silica column-based systems NucleoSpin Gel and PCR Clean-up Kit, (Macherey-Nagel, Dueren, Germany). Purified PCR products were subjected to bidirectional Sanger sequencing using the BigDye Terminator v3.1 Cycle Sequencing Kit (Thermo Applied Biosystems). Cycle sequencing reactions were performed according to standard protocols, incorporating fluorescently labeled dideoxynucleotides for chain termination. Sequencing products were processed using the BigDye XTerminator purification kit (Thermo Applied Biosystems) to remove unincorporated dyes and salts, thereby improving signal clarity. Capillary electrophoresis was carried out on the SeqStudio Genetic Analyzer (Thermo Applied Biosystems) mostly on the Medium Run module except for PCR products of exons 11–12 and 15 that were performed on the Long Run module. Raw sequence data were analyzed using dedicated software (Chromas 2.6.6 Technelysium, AU) and aligned against the wild-type reference *PKD2* sequence (NM_000297) to identify single-nucleotide variants, insertions, and deletions (NovoSNP 3.01 software, University of Antwerp, BE) [26]. Variant interpretation followed established guidelines, incorporating population databases, in silico prediction platforms (Varsome v.13.16.1 and Franklin Genoox Version 95) and bioinformatics tools where appropriate. Proper HGVS nomenclature of positive findings was checked with the Mutalyzer 3 online software (https://mutalyzer.nl/ (accessed on 30 June 2026)).

### 2.3. Multiplex Ligation-Dependent Probe Amplification (MLPA)

To complement Sanger sequencing and detect large genomic rearrangements not identifiable by conventional sequencing alone, MLPA was performed using the SALSA MLPA Probemix P352 (MRC, Holland, Amsterdam, The Netherlands) targeting both *PKD1* and *PKD2* genes. MLPA reactions were conducted according to the manufacturer’s instructions, including probe hybridization, ligation, and amplification steps. Fragment separation was achieved by capillary electrophoresis on SeqStudio, and dosage analysis was performed using Coffalyser software version 240129 (MRC, Holland), allowing detection of exon-level deletions or duplications.

### 2.4. Statistics

Results were assessed in a descriptive fashion; due to the limited patient size, no statistical result with adequate power could be reported.

## 3. Results

This pilot study provides the first in-country assessment of *PKD2* variation in a Greek laboratory setting, albeit in a small, highly ascertained cohort (*n* = 6). Pathogenic mutations were identified in 2/6 ADPKD patients (33%), plus another variant of unkwown sighnificance (VUS) in one patient markedly exceeding the ~15% detection rate typically reported for the *PKD2* gene. This apparent enrichment should be interpreted cautiously, as it is almost certainly influenced by sample size and referral bias rather than true ADPKD population-level variation.

**Figure 1:**
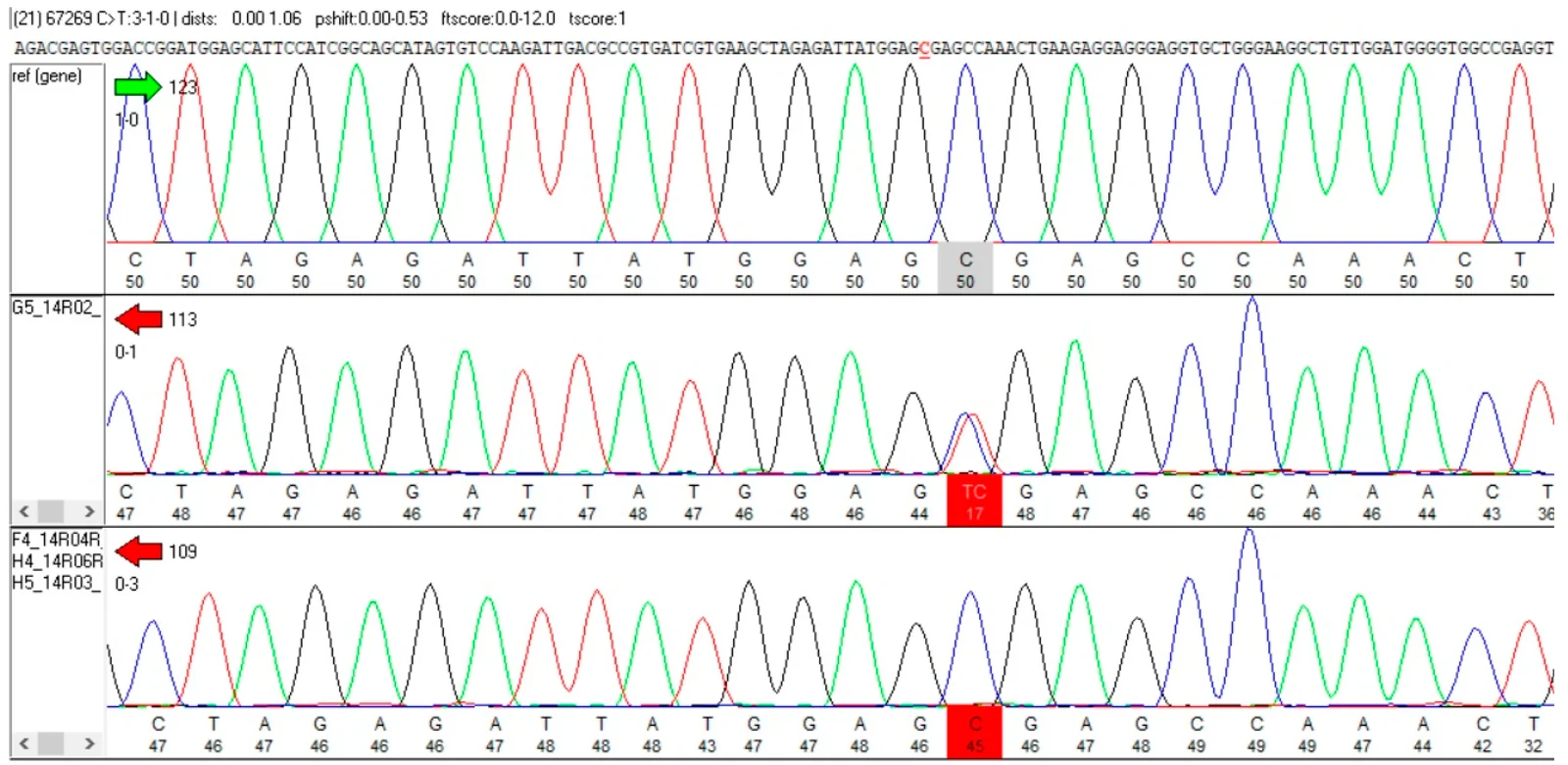
DNA sequencing analysis for the R02 ADPKD patient sample with the exon 14 single-base nonsense pathogenic *PKD2* p.Arg872Ter mutation in the middle row, indicated by red boxed TC (Novosnp 3.01 software comparison with the wild-type *PKD2* sequence in the top row and another sample in the bottom row).

**Figure 2:**
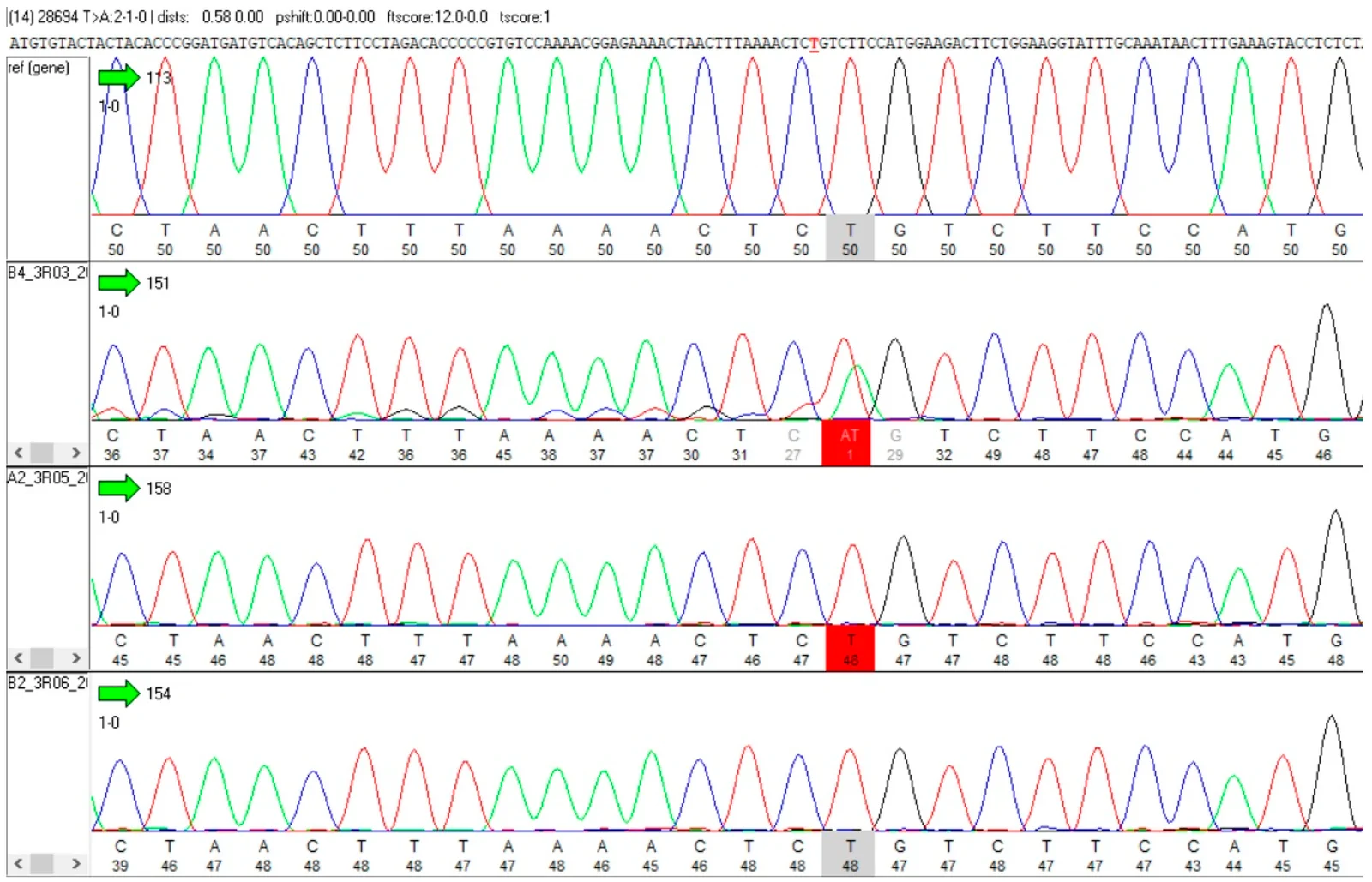
DNA sequencing analysis for the R03 ADPKD patient sample with the exon 3 single-base *PKD2* p.L273Q VUS in the second row, indicated by red boxed AT (Novosnp 3.01 software comparison with the wild-type *PKD2* sequence in the top row and other two samples in the last two rows).

The detected variants span distinct mutational classes. One patient carried the recurrent truncating nonsense variant p.Arg872Ter, a well-established hotspot and the most frequently reported pathogenic *PKD2* mutation (21 times) in the ADPKD Variant Database (https://pkdb.mayo.edu/welcome?apkd_mode=PROD (accessed on 30 June 2026)), reinforcing its clinical relevance (Figure 1). Another patient harbored three missense variants; two were previously reported as likely benign (p.R28P and P.A190T, recorded nine and 10 times respectively) and a third was a novel p.Leu273Gln ((GRCh 38, 4:88,036,328, g.33661T>A, c.818T>A) p.L273Q). This alteration was classified as VUS by the prediction platforms Varsome and Franklin Genoox based on the extremely low frequency in gnomAD populations (PM2 criterion) and on in silico prediction tools (PP3 criterion; PROVEAN and CADD scores were −3.834 and 28.5, respectively), without functional or segregation support, and therefore remains provisional (Figure 2).

**Figure 3:**
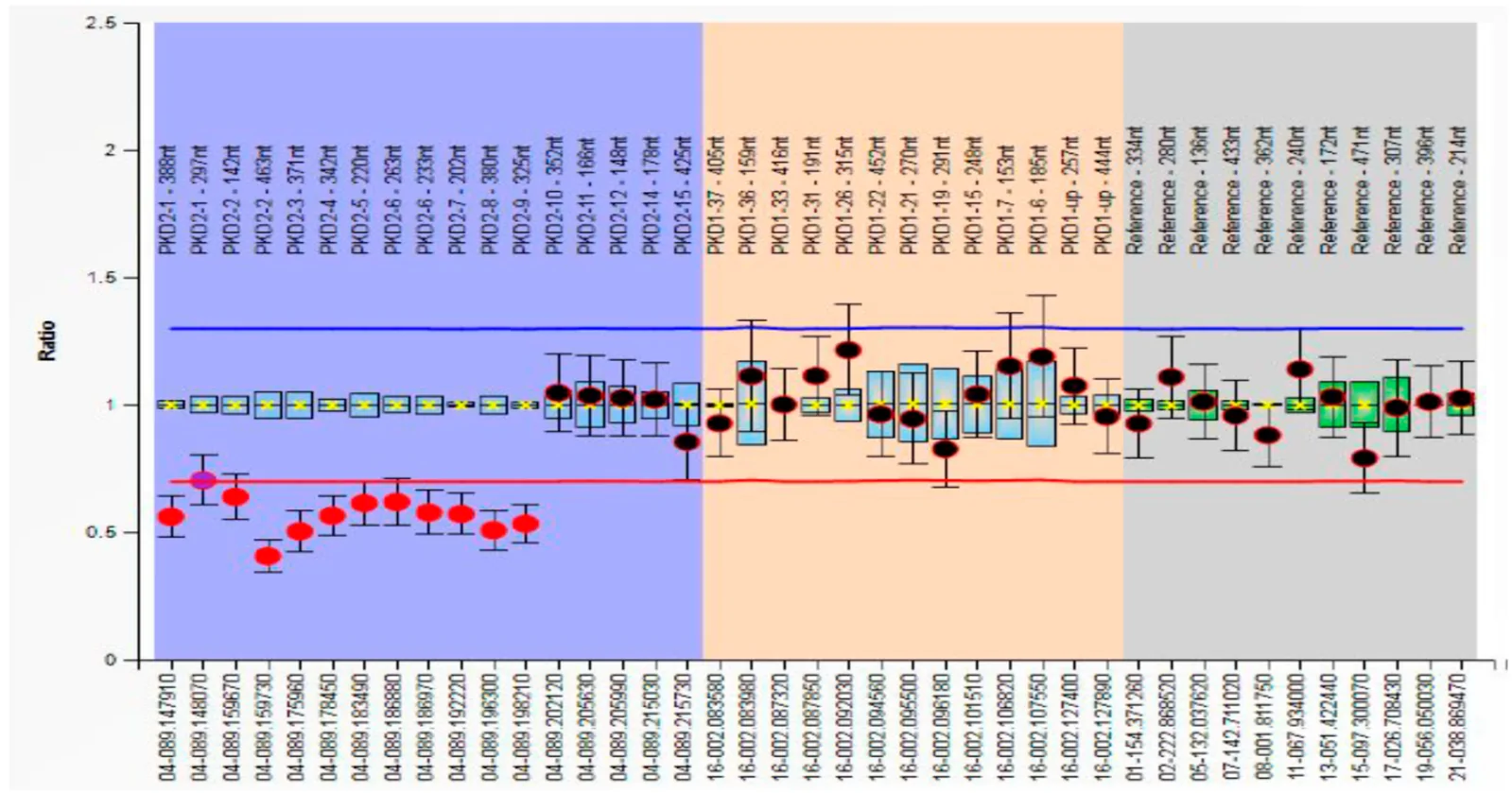
MLPA diagram of probe ratio for the R06 ADPKD patient sample (Coffalyzer software, MRC Holland). The light blue area depicts the ratio evaluation of the *PKD2* probes, the yellow zone the *PKD1* probes and the gray zone the control probes in other chromosomes. Signals less than 0.70 map a large deleted genomic area between exons 1 and 9 (red circles) that is clearly pathogenic. All other signals are considered normal (ranging between 0.70 and 1.30; red and blue horizontal lines). Details for the probes shown in the x-axis of the diagram (underneath and above) are provided in Supplementary Table S1.

In contrast, a third patient carried a previously unreported multi-exon deletion (exons 1–9), identified only through MLPA (Figure 3). This finding is particularly notable, as such structural variants are systematically missed by sequencing-only approaches and may represent an underappreciated mechanism of *PKD2* pathogenicity. The use of complementary methodologies—Sanger sequencing alongside MLPA—enabled detection of both sequence-level and copy number variants, maximizing diagnostic yield. The identification of a large genomic rearrangement in this limited cohort underscores the non-trivial contribution of structural variation and argues strongly for the routine inclusion of copy number analysis in *PKD2* testing pipelines.

All three patients with positive *PKD2* findings possessed extensive family history with three or four known PKD cases spanning three generations and having multiple liver cysts additionally, but with no aneurysms (Table 1).

The two *PKD1* patients and the two healthy subjects were free of any *PKD2* DNA alteration.

## 4. Discussion

Autosomal dominant polycystic kidney disease (ADPKD) affects approximately one in 1000 individuals and remains the leading hereditary cause of end-stage renal disease (ESRD) worldwide [5,6,7,8]. Its clinical heterogeneity reflects underlying genetic complexity, with the majority of cases attributed to *PKD1* (~85%) and *PKD2*, while additional loci contribute marginally [5,17,18]. Historically, clinical management has focused on prognostication of renal decline—primarily through estimated glomerular filtration rate (eGFR) and total kidney volume (ht-TKV)—despite emerging evidence linking ADPKD to broader complications, including aneurysms, liver cysts, and diverticulitits [8,9,11]. In parallel, the role of molecular diagnostics is shifting from an optional adjunct to a central component of precision nephrology [17,18,22,23].

Against this background, our findings provide a focused but informative signal. Pathogenic *PKD2* variants were identified in 2/6 individuals (33%) plus an additional novel VUS alteration, a detection rate that markedly exceeds the ~12–15% typically reported. This enrichment is unlikely to reflect true population frequency and should be interpreted conservatively, given the small cohort size and probable referral bias. Nevertheless, the nature of the detected variants—rather than their frequency alone—offers meaningful insights.

The observed spectrum spans recurrent, novel, and structurally complex alterations. The identification of the truncating variant p.Arg872Ter, a well-established mutational hotspot and the most frequently reported *PKD2* variant in the Mayo Clinic database, reinforces the consistency of known pathogenic mechanisms across populations [1,2,3,4,5,6]. In contrast, the novel missense variant p.Leu273Gln (p.L273Q) illustrates the persistent interpretative challenge posed by variants supported only by in silico prediction, underscoring the continued need for functional and segregation data to refine pathogenic classification [17,18]. In this patient, no segregation data could be obtained due to the loss of progenitors.

Most notably, the detection of a previously undescribed multi-exon deletion (exons 1–9), identifiable only through MLPA, highlights a critical but under-recognized dimension of *PKD2* genetics: the contribution of structural variation [17,18].

This latter finding has direct methodological and clinical implications. Structural variants of this type are systematically missed by sequencing-only approaches, including many NGS pipelines, and may therefore contribute to underdiagnosis [17,18]. This is particularly relevant in older ADPKD cohorts, where *PKD2*-associated disease—typically milder and slower progressing than *PKD1* disease—is relatively underrepresented, and where unresolved cases after standard sequencing may conceal copy number alterations [5]. In the ADPKD Variant Database, only six large *PKD2* large rearrangements have been deposited (out of a total of 196 *PKD2* pathogenic alterations, 3%).

Our data, albeit limited, provide concrete evidence that such variants are not merely theoretical but detectable contributors to disease. More broadly, these observations intersect with well-recognized technical limitations in ADPKD genotyping. While NGS has transformed molecular diagnostics, challenges remain substantial—particularly for the *PKD1* gene, due to its large size, high GC content, and the presence of six highly homologous pseudogenes, which complicate accurate read mapping and variant calling [17,18]. Even in the *PKD2* gene, which is technically more tractable, standard approaches may fail to capture structural variation. As a result, a negative sequencing result does not equate to absence of genetic causality and must be interpreted within the constraints of the applied methodology.

Collectively, our combined approach—high-resolution Sanger sequencing complemented by MLPA—maximized diagnostic sensitivity by capturing both sequence-level and copy number variants. The identification of a large genomic rearrangement in such a small cohort underscores the non-trivial contribution of structural variants and supports the routine integration of copy number analysis into *PKD2* testing pipelines, particularly in unresolved cases [17,18,22].

Importantly, the rationale for comprehensive genetic testing in ADPKD may no longer be optional. Establishing a molecular diagnosis provides actionable information for patient counseling, including recurrence risk, inheritance patterns, and access to reproductive options such as preimplantation genetic testing [17,18,22]. Within this framework, genetic testing emerges as a critical tool for aligning surveillance strategies and therapeutic decision-making—including dialysis, transplantation, and emerging targeted therapies—with underlying biological risk. Robust prognostication in ADPKD necessitates an integrated model that combines genotypic data with clinical variables, circulating biomarkers, and imaging findings within a layered risk stratification and monitoring strategy.

Nowadays, a genotype-agnostic therapeutic intervention has gained Phase 3 clinical trial approval and wide enthusiasm: the use of farabursen, an anti-miR17 oligonucleotide —properly modified in its sugar moieties in order to avoid RNAseH action—that acts as an enhancer of both polycystin 1 and 2 expression [27,28]. However, it is probable that this generic approach might only marginally slow renal decline, similar to Tolvaptan, and further precise modalities are needed. Tolvaptan, which is a competitive vasopressin receptor V2 antagonist, is the only FDA-approved drug for slowing renal cyst growth, albeit with aquaretic and hepatic side effects.

As disease-modifying therapies and targeted interventions continue to emerge, molecular stratification is increasingly required for patient selection and trial enrollment [8,9]. Patients with missense or stop codon mutations identified by genetic testing, might be benefited by precise single-base gene editing. However, our R06 patient with the large deletion identified could use the acquired info from our study and be offered a more suitable gene therapy dose of a full *PKD2* gene. This is considered feasible due to the much smaller gene size that needs to be packaged in an adenoviral vector (~3 kb compared to ~13 kb *PKD1*) if it is administered properly [28,29,30,31,32,33]. Future therapies that have already recently emerged might also include xenotransplantion with genetically modified pig kidney transplants.

Taken together, our findings argue for a pragmatic but rigorous approach to ADPKD genetics, one that acknowledges technical limitations, integrates complementary methodologies, and prioritizes diagnostic completeness, thereby introducing patients to the benefits of pioneering tailored gene therapies [28,33]. Even within a small cohort, the detection of a structurally complex *PKD2* variant serves as a reminder that reliance on sequencing alone is insufficient—and that precision nephrology depends not only on whether we test, but on how we test.

## 5. Conclusions

Our findings challenge the prevailing assumption that *PKD2* mutations are infrequent and predominantly sequence-detectable, suggesting instead that structural variants may represent an under-recognized component of the disease architecture. Although derived from a small pilot cohort in a highly selected population in an elderly ADPKD population, this signal highlights a tangible risk of underdiagnosis when copy number analysis is omitted. Conclusively, our limited data support a tiered genotyping paradigm in ADPKD, in which sequencing is systematically complemented by MLPA to achieve complete and clinically meaningful molecular diagnosis.

## Figures and Tables

**Table 1:** Clinical data of patients with positive *PKD2* findings, pathogenic or VUS (proband and starting age on KRT are indicated; other PKD patients of the same family tree are also indicated along with their age when the family was included in the study).

Patient ID	Alteration	Alteration Type	Liver Cysts	Aneurysms	Family History (Ages of PKD Patients)
R02	p.Arg872Ter	Single base	Yes	No	Mother (78 y, KRT), son (58 y), granddaughter (40 y)
R03	p.L273Q	Single base	Yes	No	Father (75 y, KRT), daughter (50 y), grandson (35 y)
R06	Del.exons1–9	Large deletion	Yes	No	Great maternal grandmother (77 y, died), mother (82 y, KRT), son (56 y), granddaughter (21 y)

## Data Availability

The original contributions presented in this study are included in the article/supplementary material. Further inquiries can be directed to the corresponding author.

## Supplementary Materials

The following supporting information can be downloaded at https://www.mdpi.com/article/10.3390/medicina62091771/s1, Table S1. Probe names, length in nucleotides and chromosomal location in hg18 (Lot number for this MLPA reagent D1-1219).
