# Supplementary material for: Beyond Sequencing: Integrating MLPA Reveals Hidden Structural PKD2 Variants and Enhances Mutation Detection in a Highly Selected ADPKD Greek Cohort
Source: Medicina (Kaunas). 2026 Sep 15;62(9):1771. doi: 10.3390/medicina62091771 (PMC13609609; doi:10.3390/medicina62091771)
Supplement: Supplementary file 1 [file medicina-62-01771-s001.zip › medicina-4437440-supplementary.pdf]

Supplementary Table S1. Probe names, length in nucleotides and chromosomal location in hg18 (Lot number for this MLPA reagent D1-1219).

**Probe name**	**Length in nt**	**Probe chromosome-location in hg18**
PKD2-1	388	04-089,147910
PKD2-1	297	04-089,148070
PKD2-2	142	04-089,159670
PKD2-2	463	04-089,159730
PKD2-3	371	04-089,175960
PKD2-4	342	04-089,178450
PKD2-5	220	04-089,183490
PKD2-6	263	04-089,186880
PKD2-6	233	04-089,186970
PKD2-7	202	04-089,192220
PKD2-8	380	04-089,196300
PKD2-9	325	04-089,198210
PKD2-10	352	04-089,202120
PKD2-11	166	04-089,205630
PKD2-12	148	04-089,205990
PKD2-14	478	04-089,215030
PKD2-15	425	04-089,215730
PKD1-37	405	16-002,083580
PKD1-36	159	16-002,083980
PKD1-33	416	16-002,087320
PKD1-31	191	16-002,087850
PKD1-26	315	16-002,092030
PKD1-22	452	16-002,094560
PKD1-21	270	16-002,095500
PKD1-19	291	16-002,096180
PKD1-15	248	16-002,101510
PKD1-7	153	16-002,106820
PKD1-6	185	16-002,107550
PKD1-up	257	16-002,127400
PKD1-up	444	16-002,127890
Reference	334	01-154,371260
Reference	280	02-222,868520
Reference	136	05-132,037620
Reference	433	07-142,711020
Reference	362	08-001,811750
Reference	240	11-067,934000
Reference	172	13-051,422440
Reference	471	15-097,300070
Reference	307	17-026,708430
Reference	396	19-056,050030
Reference	214	21-038,869470
